# Spatial and temporal variation in harvest probabilities for American black duck

**DOI:** 10.1002/ece3.1484

**Published:** 2015-04-16

**Authors:** Christian Roy, Steven G Cumming, Eliot JB McIntire

**Affiliations:** 1 Faculté de foresterie, de géographie et de géomatique and Centre d’étude de la Forêt, Université LavalPavillon Abitibi-Price, 2405 Rue de la Terrasse, Québec, Canada; 2 Natural Resources Canada, Pacific Forestry Centre506 Burnside Road West, Victoria, British Columbia, Canada, V8Z 1M5

**Keywords:** Bands recoveries, harvest management, hierarchical logistic regression, hunting effort, spatial prediction, waterfowl management

## Abstract

Assessing spatial variation in waterfowl harvest probabilities from banding data is challenging because reporting and recovery probabilities have distinct spatial patterns that covary temporally with harvesting regulations, hunter effort, and reporting methods. We analyzed direct band recovery data from American black ducks banded on the Canadian breeding grounds from 1970 through 2010. Data were registered to a 1-degree grid and analyzed using hierarchical logistic regression models with spatially correlated errors to estimate the annual probabilities of band recovery and the proportion of individuals recovered in Canada. Probability of harvest was estimated from these values, in combination with independent estimates of reporting probabilities in Canada and the USA. Model covariates included estimates of hunting effort and factors for harvest regulation and band reporting methods. Both the band recovery processes and the proportion of individuals recovered in Canada had significant spatial structure. Recovery probabilities were highest in southern Ontario, along the Saint Lawrence River in Quebec, and in Nova Scotia. Black ducks breeding in Nova Scotia and southern Quebec were harvested predominantly in Canada. Recovery probabilities for juveniles were correlated with hunter effort, while the adult recoveries were weakly correlated with the implementation of stricter harvest regulations in the early 1980s. Mean harvest probability decreased in the northern portion of the survey area but remained stable or even increased in the south. Harvest probabilities for juveniles in 2010 exceeded 20% in southern Quebec and the Atlantic provinces. Our results demonstrate fine-scale variation in harvest probabilities for black duck on the Canadian breeding ground. In particular, harvest probabilities should be closely monitored along the Saint Lawrence River system and in the Atlantic provinces to avoid overexploitation.

## Introduction

Measuring and regulating the annual per capita rate of harvest, that is, the probability that an individual will be taken by a hunter, are essential to the management of any harvested population. In North America, waterfowl populations are monitored through an extensive banding program. The sample of banded birds is assumed to be representative of the population of interest (Nichols et al. 1995b). Harvest probability (*h*) is the probability that a duck alive at the beginning of the hunting season will be harvested. Recovered bands are those found on harvested ducks by hunters. These are meant to be reported to the authorities. Harvest probability could be estimated directly from the proportion of bands recovered (*f*) if all recovered bands were reported by hunters. As this is not the case, adjustments must be made in calculating *h* to account for the reporting probability, *λ* (Brownie et al. 1985). This probability depends on hunter behavior and can be influenced by factors such as reporting method, region, or species (Royle and Garrettson 2005; Zimmerman et al. 2009; Boomer et al. 2013). Reward band studies are typically used to estimate *λ* (Henny and Burnham 1976). The reporting probability can then be used to correct the recovery probability and derive the harvest probability (*h* - *f* /*λ*). Because many spatially varying factors can affect *f* and *λ*, *h* is also thought to vary spatially. Understanding this variation, and the factors giving rise to it, is important to the management and conservation of harvested species (Williams et al. 2002).

The American black duck *Anas rubripes* (Brewster) is of concern to waterfowl managers because continental populations declined markedly between the 1950s and 1990s (Geis et al. 1971; Longcore et al. 2000b; Devers and Collins 2011). In response to this decline, the United States Fish and Wildlife Service (USFWS) and the Canadian Wildlife Service (CWS) changed their harvest regulations in 1983 and 1984, respectively, imposing harvest restrictions intended to reduce harvest by 25% (Devers and Collins 2011). While the continental population size is now stable, it remains below the objectives of the North American Waterfowl Management Plan (NAWMP; USFWS 2012). There is also substantial regional variation in population trends and productivity (Longcore et al. 2000b). Populations wintering in the northeastern US seem to be stable or increasing, whereas those wintering in areas associated with the Mississippi flyway are still declining (Link et al. 2006). Although the precise role of harvest rates in black duck population dynamics is still debated (Merendino et al. 1993; Conroy et al. 2002; Link et al. 2006; Maisonneuve et al. 2006; Brook et al. 2009), harvesting is the only source of mortality that managers can actively control.

**Figure 1:**
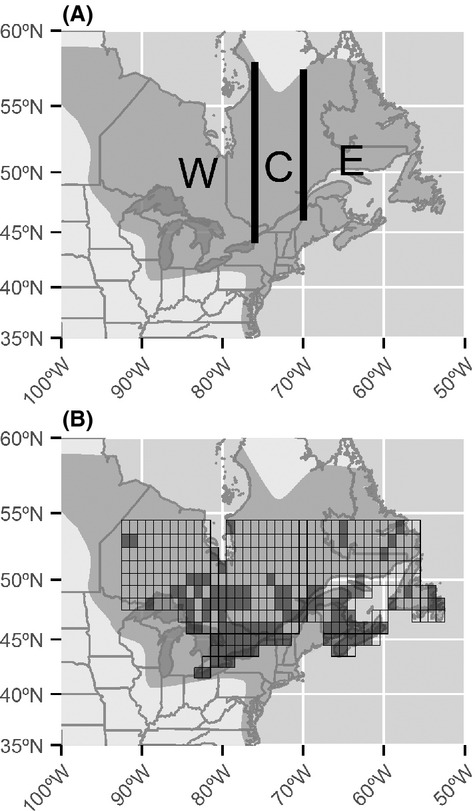
(A) Black duck breeding distribution according to Nature Serve (dark gray), with breeding and harvest area delineations used for management (vertical lines). The management zones in Canada are the Western (W), central (C), and eastern (E) breeding and harvest areas; (B) Spatial grid used for prediction. Shaded cells represent the 1-degree banding blocks where black ducks were banded during at least one year over the period 1970–2010.

The estimation of *h* for black duck is complicated by several factors. Within the Canadian breeding zone, a considerable proportion of the hunting effort is concentrated along the Saint Lawrence River system and in the Atlantic Provinces. This suggests that hunting pressure on local populations may be disproportionately intense in the south and east of the breeding zone (Reed and Boyd 1974; Parker 1991; Longcore et al. 1991, 2000a; Cousineau et al. 2014). The location of harvest, measured as the probability that a harvested duck will be harvested in Canada (or in the USA) also seems to vary spatially (Conroy et al. 2005). Ashley et al. (2010) used stable isotope techniques to assess the origin of wings collected by the CWS Species Composition Survey. They found that northern boreal populations were poorly represented in their sample and suggested that individuals from the boreal populations might overfly southern Canada, to be harvested in the United States (Ashley et al. 2010). In a similar study, Cousineau et al. (2014) demonstrated that the black duck harvest in Quebec is heavily biased toward the populations breeding in the agricultural regions of southern Quebec. Estimating *h* is further complicated by differences in λ among the two national jurisdictions (Garrettson et al. 2013), and by differential, decreasing trends in hunting effort (Vrtiska et al. 2013). Given spatial variation in *h*, black duck breeding stocks should be managed individually in an adaptive management paradigm (Conroy et al. 2002). However, due to knowledge limitations and management constraints, managed populations are defined according to migration corridors and political jurisdictions (Fig.1A; Black Duck Joint Black Duck Joint Venture 1992; Zimpfer and Conroy 2006). Our objective was to estimate spatially and temporally explicit variations in *h*, corrected for variation in *λ*, and to test whether the 1980s changes in regulations were effective in decreasing *h*. To do so, we had to (A) estimate spatial variation in *f* for black ducks, and estimate the effects of post-1983 harvest restrictions and the decline in hunting effort on *f*; (B) estimate how the proportion of black ducks recovered in Canada (*P*_CAN_) versus in the USA (*P*_USA_) varies spatially and temporally. For estimation, we used hierarchical Bayesian models with spatial and temporal random effects.

## Methods

### Banding data

We obtained black duck banding and recovery data for the years 1970 to 2010 (*n* - 41) from the United States Geological Survey's Bird Banding Laboratory (BBL, Laurel, Maryland). At the time of banding, ducks were sexed and aged as either juvenile (hatch-year birds) or adult (after hatch-year). Locations of banding, release, and recovery were registered to 10 arc-minute blocks. We included normal wild birds released in the same block as the banding location, control bands from rewards programs, and birds that were captured with night lighting techniques in the analysis. We excluded reward bands and bands solicited through mail surveys. We selected banding events that took place in Ontario, Quebec, and the Atlantic Provinces (Fig.1A) during the preseason banding period (July to September) between 42°N and 57°N. We restricted the analysis to birds that were harvested during the hunting season (September to February) immediately following the banding period (i.e., direct recoveries). Thus, we required no assumptions about interyear fidelity to the spatial locations and avoided any bias that could be introduced by survival rates.

We grouped banding and recovery events into “banding blocks” of size 1° longitude by 1° latitude (Fig.1B) as it is the nominal spatial resolution available for the BBL band-recovery database (Royle and Dubovsky 2001). We determined the number of individuals banded in and recovered in each block and year. For each cell, we stratified band counts by age, because juveniles are more vulnerable to harvest than adults (Reynolds 1987; Longcore et al. 2000b). We also stratified by band type because technological change during the survey period made reporting bands easier, thus increasing λ (Nichols et al. 1995b; Guillemain 2010; Padding and Royle 2012). We defined four band types: “avise,” “zip code,” “toll-free,” and “web address”. Prior to 1993, standard leg bands were inscribed “AVISE BIRD BAND WRITE WASHINGTON DC USA.” After 1993, the inscriptions on some bands were modified to include a United States postal zip code, to make reporting easier. The next important change was made in 1995 when the BBL introduced a phone line to make bands reporting more convenient. As a result, “CALL 1-800-327-BAND” was added to the bands and these new “toll-free” bands were incrementally implemented in 1996 for black ducks. The standard bands were modified again in 2007 after the implementation of a dedicated website, and the web address replaced the postal address. These bands were deployed in limited numbers on black ducks in 2007 and in the subsequent years. Overall, λ increased in the USA following the implementation of the toll-free number and the web-address bands (Royle and Garrettson 2005; Padding and Royle 2012; Sanders and Otis 2012). For black duck in particular, λ more than doubled between 1984 and 2002 (Conroy and Blandin 1984; Garrettson et al. 2013). We expected that this increase would be explained by the changes in the reporting system. While annual λ are basically nuisance parameters in modeling *h,* they need to be accounted for because they affect the estimation of *f*. We did not consider interactions between band type and sex because black ducks are sexually monomorphic such that hunters cannot reliably determine the sex of individuals in flight (Metz and Ankney 1991; Wilson and Rohwer 1995). Moreover, λ for male and female black ducks do not differ significantly (Garrettson et al. 2013).

Our modeling strategy was divided in 3 steps. To estimate the harvest probabilities, we need to divide the estimated recovery probabilities by the reporting probabilities (*h - f/λ*). Our first step was to model *f* per banding block. As it has recently been shown that λ for black ducks varies in function of jurisdiction (Garrettson et al. 2013), we also required to estimate the proportion of ducks recovered in each jurisdiction ( *P*_CAN_ and *P*_USA_) at the block level to estimate *h* accurately. Our second step was therefore to model the proportion of black duck recovered in Canada (*P*_CAN_). Our third and final step was to estimate *h* at the scale of the banding block by combining our estimated *f* and the estimated proportions of bands recovered in each jurisdiction (*P*_CAN_ and *P*_USA_) with previously reported λ by Garrettson et al. (2013).

### Recovery probability

We expected that the implementation of more restrictive hunting regulations would cause a decrease in *h* that should be reflected in *f*. To account for changes in hunting regulations, we included an annual categorical variable with a change point at 1983, the year when new regulations began to be implemented (Francis et al. 1998; Devers and Collins 2011). Concurrent with much of the period of population decline, and the regulatory change, was a long-term decline in hunter participation in both Canada and the USA (Vrtiska et al. 2013). In the USA, mean annual duck-stamp sales declined 36% relative to the 1970s, while, in Canada, the numbers of hunters declined by 72% between 1978 and 2007 (Vrtiska et al. 2013). An important difference, however, was that the number of American hunters increased during the 1990s, while the number of Canadians hunters continued to decline. We expected that *h* would be correlated with participation, or hunting effort. To measure hunting effort, we obtained annual sales figures of Canadian migratory bird hunting permits (Environment Canada 2013) and of US duck stamps (Robert Raftovich, USFWS unpublished data). We summed the annual counts of permits from Ontario, Quebec, and the Atlantic Provinces and of duck stamps sold in the Atlantic and Mississippi flyways as a surrogate for total annual hunting effort. Finally, Henny and Burnham (1976) suggested that higher banding effort would reduce λ should hunters, frequently encountering bands from the same location, lose interest in reporting them. To test for, and account for, this effect, we also included the total numbers of black duck banded during the year as a covariate.

Our approach to modeling recovery probabilities is a temporal extension of Royle and Dubovsky's (2001) model of spatial variation in *f*. The number of direct recoveries *y*_*i,t*_ from banding block (Fig.1B) *i* in year *t* was modeled as a binomial random variable.

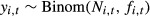
1

where *N*_*i,t*_ is the number of banding events and *f*_*i,t*_ is the recovery probability, both indexed by location and year. We modeled *f* by mixed-effects logistic regression (eqs 1 and 2), following:

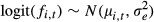
2

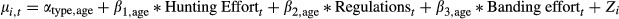
3

where *μ*_*i,t*_ is the fitted mean proportion of recoveries on the logit scale and 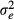 is the small-scale heterogeneity parameter to accommodate random departures from this mean. In the systematic component (eq. 2), *α* is the mean recovery probability by age class and band type, *β*_k,age_ are age-class-specific coefficients for the three covariates, and *Z*_*i*_ is a spatially autocorrelated random site effect.

The random site effect was modeled as a multivariate normal distribution (eq. 3) where 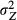 represents the spatial variance and **K** is an *n* by *n* matrix specifying the correlation structure among the *n* banding blocks. We specified an exponential correlation function (eq. 4) for the correlation matrix **K**, where each element of the matrix (*ρ*_*i,j*_) represents the correlation between banding blocks *i* and *j*; *d*_*i,j*_ is the distance in km between block centroids; and *θ* is the range parameter that models the decay of correlation with distance:

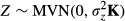
4

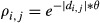
5

We chose an exponential correlation function as there is generally little justification for more complicated forms in ecology (Royle et al. 2002). For spatial exponential models, the value of the range is often difficult to interpret by itself, so we report instead effective range (≈ (*θ/*3)^−1^) that is the distance at which the spatial correlation falls below 0.05 (Banerjee et al. 2004). We included a spatial effect because we expected that black duck population along the Saint Lawrence River system and in the Atlantic Provinces would be harvested at higher rates than elsewhere, because of higher local hunting pressure. The spatial random error and small-scale heterogeneity parameter were assumed constant among age classes.

### Proportion of recoveries in Canada

We expected the decreasing trend in hunter effort over time would diminish the proportion of black ducks harvested in Canada (*P*_CAN_) because the decline was greater in Canada than in the USA (Environment Canada 2013; Vrtiska et al. 2013). However, we expected the changes in regulation to increase this proportion, because they were more restrictive in the USA. Finally, we expected *P*_CAN_ to diminish as a function of the implementation of the toll-free numbers and the web-based reporting application. We expected this because recent changes in reporting probabilities indicate that American hunters are more likely than Canadians to report bands via the new methods (Garrettson et al. 2013). We expected that *P*_CAN_ would be greater along the Saint Lawrence River system and in the Atlantic Provinces than in Northern Quebec and Northwestern Ontario because of the higher hunting pressure in these regions. We used age-class-specific intercepts and coefficients for these terms (eq. 2) as we expected juveniles to be more vulnerable than adults to hunting and thus have a greater likelihood of being harvested in Canada compared to an adult banded in the same black and year.

The number *x*_*i,t*_ of bands from banding block *i* in year *t* recovered in Canada was modeled as a binomial random variable.

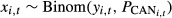
6

where *y*_*i,t*_ is the total number of direct recoveries of bands for the banding block *i* at time *t* and 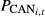 is the estimated probability of bands being recovered in Canada. We modeled this probability using a mixed-effects logistic regression. We used a similar approach as in the model of recovery probability to assess small-scale heterogeneity, the effects of explanatory variables and the random site effect (eq. 6 & 7). For this model, we expected the probability of black ducks being reported harvested in Canada would be greater along the Saint Lawrence River system and in the Atlantic Provinces than in the Northern Quebec and Northwestern Ontario because of the higher hunting pressure in these regions. The spatial random effect was modeled as in equations 3 and 4.

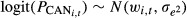
7

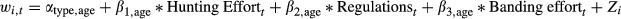
8

We derived the proportion of bands recovered in the USA as 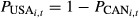


### Estimation

We used hierarchical Bayesian methods to fit the models. For the intercepts, we used a blocking strategy where four pairs of intercepts were drawn from a bivariate normal distribution. Members of a pair represented the two age groups within each band type. We used a separation strategy for the priors on the variance–covariance matrix of the bivariate distribution. We used a prior based on C-vines approach as defined in Lewandowski et al. (2009) with a uniform prior for the correlation matrix, and we used a half-Cauchy distribution with a mean of 0 and a scale of 5 for both standard deviations. We used normal priors on the effort covariates (*β*_*k*_) with a mean of zero and a standard deviation of 5, truncated to (−20, 20). Following Gelman (2006), we used half-Cauchy priors for standard deviation parameters (*σ*_e_,*σ*_Z_). We used a location value of 0, a scale of 1, and truncated the distribution between 0 and 20. For the range parameter (*θ*) in the spatial random effect, we used a uniform prior. We used the information available in the distance matrix to determine the range of the prior. We used the maximum distance (2756 km) in the distance matrix to determine the lower bound (− log[0.99]/2756) of the distribution and the minimum (63 km) distance to determine the upper bound (− log[0.01]/63). The minimum value of *θ* corresponds to a correlation of 0.99 between the banding blocks at the maximum distance in the study area, while the maximum value of *θ* corresponds to correlation of 0.01 between the banding blocks at the minimum distance. Spatial models can be plagued by identifiability issues between the mean and the spatial random effect. A common solution is to use a hierarchical centering reparameterization (Gelfand et al. 1995). This option was not available to us, because the spatial effect is shared by the juveniles and adults. We resolved the issue by constraining *Z* such that 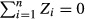 (Rue and Held 2005). This is consistent with the assumption that *E*[Z_*i*_] - 0 and also makes interpreting the spatial errors straightforward. Banding blocks taking values less than or greater than zero have lesser or greater than mean probabilities, respectively.

We ran six chains and used a burn-in of 250 iterations that we discarded followed by 1250 iterations. Convergence was assessed using the split scale reduction factor R (Gelman et al. 2013) and visual inspection of the plotted chains. Models were estimated using Hamiltonian Monte Carlo (HMC) sampling, as implemented in Stan version 2.3 software accessed via the rstan package (Appendix S1; Stan Development Team 2013) in R (R Core Team 2013). Stan implements the “No U-turn” sampler (Hoffman and Gelman 2014), which automatically optimizes the parameters of the HMC sampling. We made our predictions on a grid consisting of 335 banding blocks (Fig.1B).

### Derivation of harvest probabilities

For comparison, we calculated harvest probabilities before the implementation of the changes in regulations (1982) and for the most recent period (2010). For the period before the changes in regulations, we used the reporting probabilities reported by Conroy and Blandin (λ - 0.43; 1984). Because the authors did not report significant spatial variation, harvest probabilities could be calculated straightforwardly as 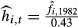 where 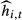 is the estimated harvesting probability in banding block *i* in year *t*, 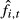 is the estimated recovery probability. For the most recent period, we used regional reporting probabilities (*λ*_CAN_ - 0.50 and *λ*_USA _- 0.73; Garrettson et al. 2013), as follows:

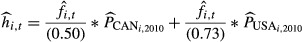
9

To assess the importance of the change in harvest probabilities, we calculated the risk ratios 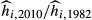. Ratios greater than one indicate an increase in harvest probabilities.

## Results

### Recovery probability

**Figure 2:**
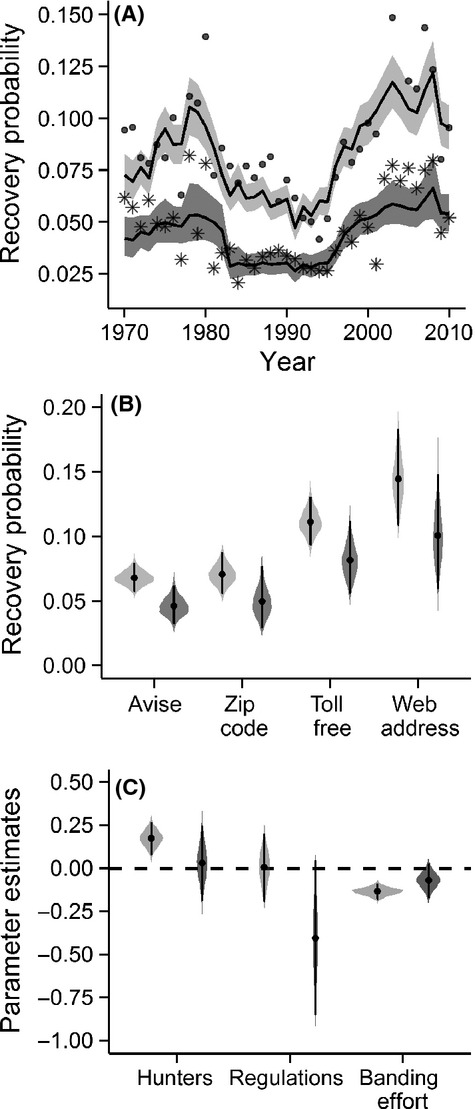
Temporal variation in annual band recovery probability (eqs 1,2,3). (A) model predictions (black line) with 95% credible intervals (CIs; shaded area), and observed proportions of direct recoveries for juveniles (●) and adults (*). Predictions were weighted in function of the proportion of each band type in the sample for a given year; (B) violin plots of full posterior distribution of the estimated coefficients (eqs 2) for band type and (C) for other model covariates. Light gray represent juveniles while dark gray represent the adults.

We analyzed recoveries of 97,292 black duck bands (77,585 juveniles and 19,707 adults) from 105 banding blocks. Annual totals of bands applied ranged from 1014 to 4142 (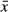 - 2 372, SD - 733). The annual totals banded decreased temporarily in the late 1970s to early 1980s and were stable thereafter. There were 7 445 direct recoveries (6 563 juveniles and 882 adults) from 89 banding blocks. Estimated recovery probabilities increased slightly during the 1970s, before declining to a stable, but low level from the early 1980s until the late 1990s after which they recovered to the levels observed during the 1970s (Fig.2A). For the avise bands, the juvenile mean recovery probability was 0.068 (95% Credible Interval - 0.057–0.079; Fig.2B) and 0.046 (95% CI - 0.032–0.061) for the adults. Adding the zip code to the bands did not increase the mean recovery probability for either juveniles (0.071; 95% CI - 0.055–0.086) or adults (0.050; 95% CI - 0.028–0.074). However, adding the toll-free line numbers to the bands increased the recovery probability for juveniles (0.111; 95% - 0.093–0.130) and adults (0.082; 95% CI - 0.054–0.109). Adding the website address to the bands further increased the mean recovery probability for juveniles (0.101; 95% CI - 0.057–0.145) but not for adults (0.145; 95% CI - 0.107–0.182). The mean recovery probability tended to be higher for juveniles than for adults for all band types, but the difference was significant only for avise bands (Fig.2B). Our power to detect differences among the age classes within the other band types was limited, because the avise bands represented 67% of the data set.

**Figure 3:**
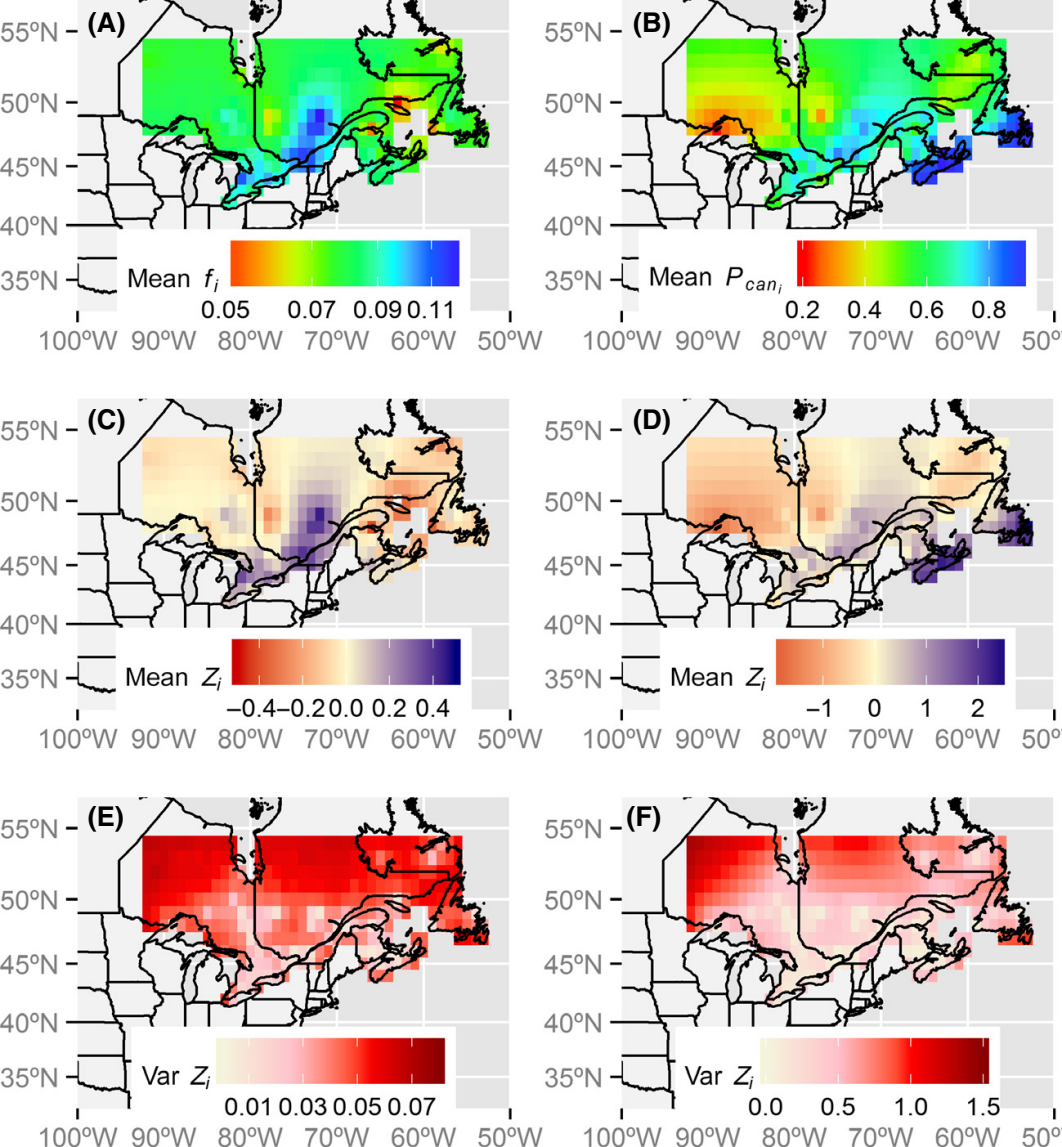
Spatial predictions of recovery probabilities (left column) and proportion of bands recovered in Canada (right column) for juvenile black ducks. Predicted probabilities by banding block (A, B); mean random effect for sites (C,D); variance associated with the site effect (E, F).

**Figure 4:**
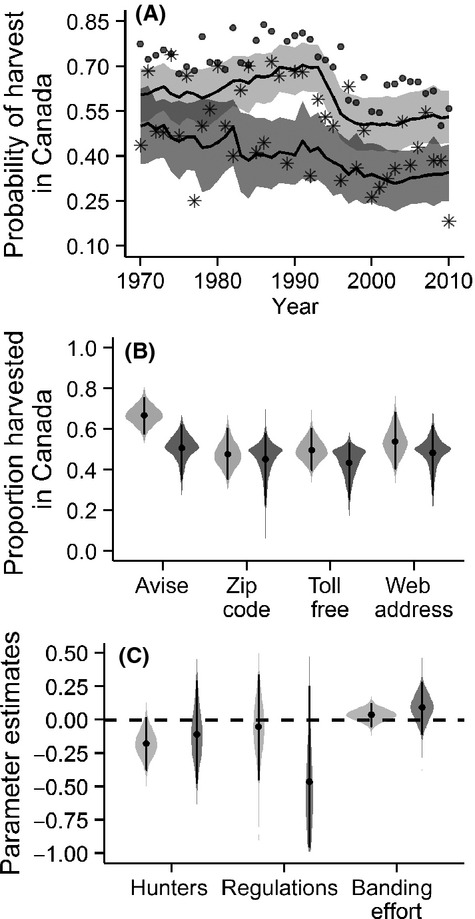
Temporal variation in the proportion of recoveries in Canada (eqs 6,7). (A) model predictions (black line) with 95% credible intervals (CIs; shaded area), and observed proportions of direct recoveries for juveniles (●) and adults (*). Predictions were weighted in function of the proportion of each band type in the sample for a given year; (B) violin plots of full posterior distribution of the estimated coefficients (eqs 7) for band type and (C) for other model covariates. Light gray represents juveniles while dark gray represents the adults.

Hunting effort had a positive effect on juvenile recovery (0.175; 95% CI - 0.082–0.270; Fig.4C) but not on adult recovery (0.032; 95% CI - −0.178–0.252). We did not detect an effect of regulation for juveniles (0.008; 95% CI - −0.188–0.205), but there was a strong tendency for the recovery probabilities of adults to decrease after the implementation of the new regulations (−0.409; 95% CI - −0.881–0.021). Recovery probability decreased significantly with banding effort for juveniles (−0.133; 95% CI - −0.179 to −0.092) and also had a tendency to decrease for adults (−0.069; 95% CI - −0.165–0.028). The spatial 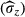 - 0.267; 95% CI - 0.172–0.387) and heterogeneity 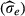 - 0.300; 95% CI - 0.259–0.341) standard deviations were similar in magnitude, suggesting that spatial variation in band recovery was limited. Blocks separated by more than 462 km (172–3212 km) were correlated at less than 0.05. However, the spatial standard deviation and the range parameters are not uniquely identifiable in the model. Individual inference for these parameters should therefore be treated carefully. As expected, the distribution of spatial random effects revealed a pattern of elevated 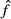 for individuals banded in southern Ontario, in the Saguenay–Lac St-Jean region of Québec, along the Saint Lawrence River, and in Nova Scotia (Fig.3A and B). The uncertainty in the estimated spatial random effects increased markedly in northern areas, particularly in northwest Ontario, owing to small sample sizes (Fig.3C).

### Proportion of recoveries in Canada

The predicted proportion of black ducks recovered in Canada declined in the middle of the 1990s and remained stable during the 2000s (Fig.4A). The model systematically underpredicted the response for juveniles. This is probably because an overwhelming proportion of the observed data are associated with banding blocks located in the south. The mean proportion of juvenile black ducks recovered in Canada with the avise bands was 0.668 (95% CI - 0.578–0.756; Fig.4B). This proportion decreased with the implementation of the zip code bands (0.474; 95% CI - 0.358–0.608), but remained stable after the implementation of the toll-free bands (0.496; 95% CI - 0.395–0.603). The implementation of the web-address bands point to an increase in the proportion of bands reported in Canada (0.537; 95% CI - 0.397–0.677), but the increase was not significant. The mean proportion of adult black ducks recovered in Canada with the avise bands was 0.507 (95% CI - 0.372–0.635), and remained stable after the implementation of the three new band types (Fig.4B). The mean response tended to be higher for juveniles than for adults for all band types, but was significantly so only for the avise bands (Fig.4B). However, the limited amount of direct adult recoveries (*n* - 882) class limited our capacity to detect the effect of band type on this age class.

Hunting effort tended to decrease the proportion of juvenile black ducks recovered in Canada (−0.179; 95% CI - −0.386–0.010; Fig.4C), but the effect overlapped zero using 95% CI. The changes in regulation had no discernible effect on the response. Banding effort tended to increase the proportion of black duck recovered in Canada, but the 95% credible interval overlapped zero for both juveniles and adults. The spatial standard deviation 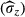 - 1.367; 95% CI 0.724–2.389) was 2.72 times larger than the heterogeneity standard deviation 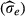 - 0.502; 95% CI 0.384–0.619) suggesting that spatial processes were of greater importance in this model than in the recovery probability model. Blocks separated by more than 1196 km (437–9728 km) were correlated by less than 5%, which is a distance 2.6 times greater than for the recovery probabilities. However, as with the recovery probability model, the spatial standard deviation and the range parameter were highly correlated making their individual interpretation inappropriate. The relatively long-range spatial correlation corresponded to a strong geographical pattern (Fig.3B). These results imply that black ducks originating in Nova Scotia and southern Quebec were recovered predominantly in Canada, while the birds banded in northwestern Ontario and northeastern Quebec were recovered in greater proportion in the USA (Fig.3C). As with the recovery probability model, the variance in the estimated spatial random effects was greatest in the north of the study region (Fig.3F) where data were limited.

### Harvest probabilities

**Figure 5:**
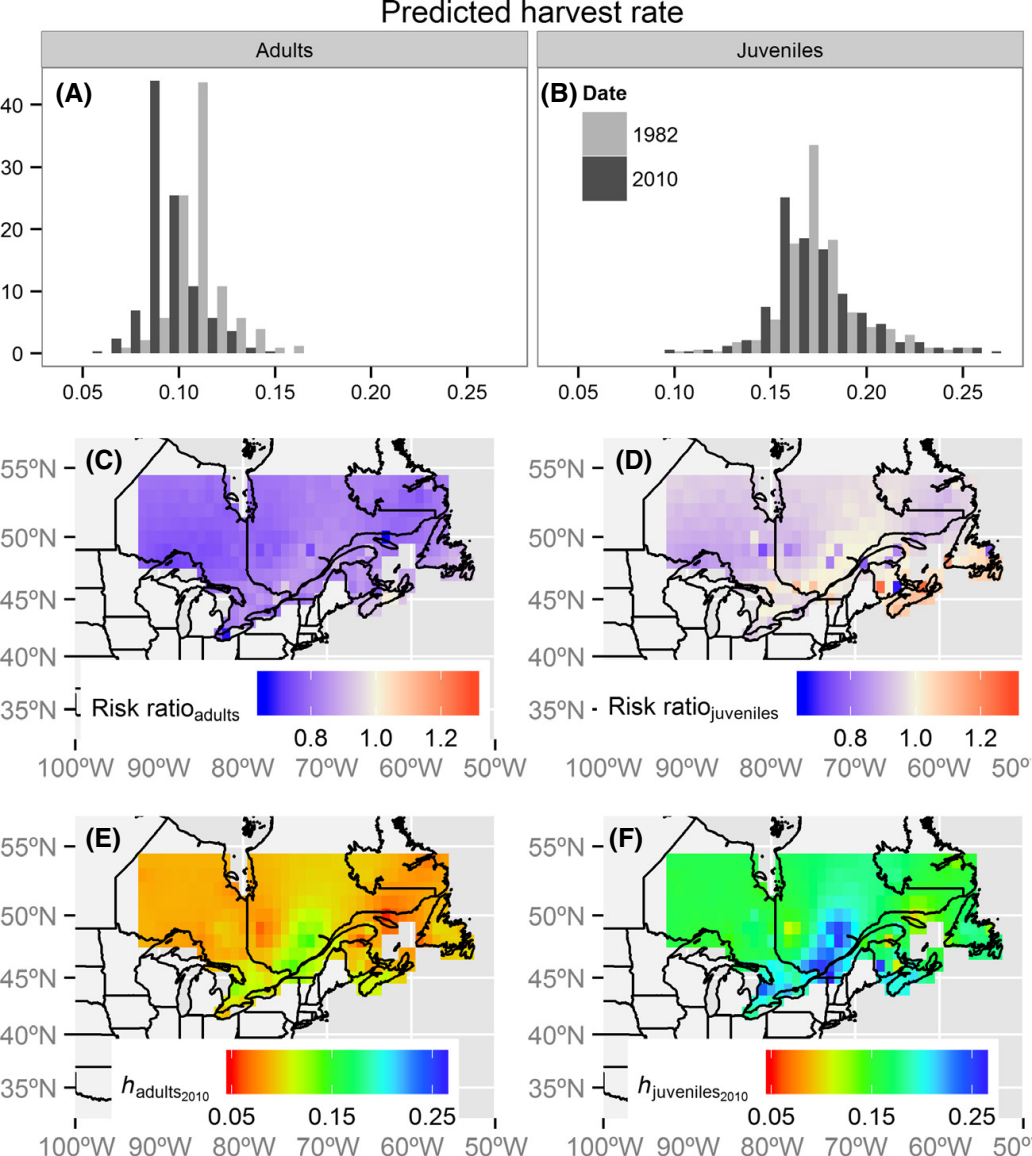
Harvest probabilities for adult (left column) and juvenile (right column) black ducks. Mean-derived harvest probability distributions before the regulation changes (1982) and during the most recent hunting period (2010) (A, B); risk ratio between the probabilities of harvest in 2010 and 1982 (C, D); predicted harvest probabilities (eq. 1) for adult and juvenile black ducks in 2010 (E, F).

In 1982, block level 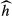 for black duck were between 0.072 and 0.167 for adults and 0.110–0.285 for juveniles (Fig.5A and B). In 2010, probabilities ranged between 0.052 and 0.141 for adults and 0.099–0.261 for juveniles (Fig.5A and B). The risk ratio in 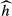 between 1982 and 2010 ranged from 0.650 to 1.001 with a mean of 0.806 for adults and from 0.664 to 1.255 with a mean of 0.952 for juveniles. Harvest probabilities decreased in the northern regions, but there were some local increases, especially in the Atlantic Provinces and along the Saint Lawrence River (Fig.5C and D). Harvest probabilities are highest in southern Quebec and in the Atlantic provinces and lowest in northwestern and northeastern populations (Fig.5E and F). The spatial pattern in 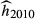 is driven mainly by spatial variation in the proportion of recoveries in Canada.

## Discussion

Given recently published estimates of λ (Garrettson et al. 2013), our work shows that 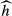 has decreased for ducks breeding in the northern region but remains high in the southern region of Canada. As of 2010, 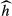 are highest in the Atlantic Provinces, along the Saint Lawrence River system in Quebec, and in southern Ontario. The pattern observed in 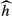 reflects marked and distinct spatial variation in both the 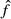 and the locations of recoveries (*P*_CAN_ vs. *P*_USA_).

### Recovery probability

Compared to adult black ducks, mean recovery probability was significantly higher for juveniles with avise bands. There was a strong tendency for higher juvenile recovery with all other band types, but the differences were not significant according to the 95% credible intervals. Higher juvenile recovery probabilities have been found for many waterfowl species (Reynolds 1987; Guillemain et al. 2010, 2013) including black duck (Reed and Boyd 1974; Parker 1991; Longcore et al. 2000a). The increased vulnerability of juveniles is typically attributed to naivety of individuals not previously exposed to hunting (Francis et al. 1992; Calvert et al. 2005). Juveniles also require more food and make more daily feeding flights than adults and consequently could have greater exposure to hunting than adults under equal degrees of hunting pressure, particularly early in the season (Martin and Carney 1977).

We found juvenile black duck 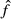 to be positively associated with hunting effort. We found only a weak effect of the 1983 change in regulations on adult 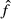. Thus, we conclude that the decline in hunting effort since the 1970s, more so than regulatory changes, helped managers reach their population objectives. However, hunting effort may be inversely related to the strength of regulation. Identifying the separate contributions of effort and regulation can therefore be difficult (Nichols et al. 1995a; Rice et al. 2010; Sedinger and Herzog 2012). Moreover, the regulations were not implemented at the same time in the US and in Canada, and the rules were modified in some jurisdictions since 1983 (Conroy and Peterson 2012). These factors could have hampered our capacity to detect the expected effects of regulations. Juveniles are predominantly harvested in Canada, where the numbers of hunters have drastically diminished. Adults are predominately harvested in the US, where the decrease in hunters was less severe. Thus, it is plausible that hunting effort has been the limiting factor for juvenile harvest rates, while adult harvest has been limited by regulations.

We also found substantial evidence that part of the increase in *f* is related to human factors that are associated with changes in λ rather than changes in *h*. The implementation of the toll-free bands in 1995 simplified the reporting process and we found increased 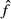 coincident with this event, which is consistent with previous studies (Royle and Garrettson 2005; Padding and Royle 2012; Garrettson et al. 2013). We also detected a positive effect of the implementation of the new web-address bands for juveniles. However, our sample size was limited for these type of bands (1 < % of all the bands) and covered only 3 years postimplementation. Sanders and Otis (2012) detected a greater λ for web-address bands for mourning dove (*Zenaida macroura*) and underlined the importance of accounting for the increasing λ in future monitoring programs.

### Proportion of recoveries in Canada

We found that 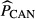 has decreased during the study period. The decrease in 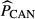 coincided with an increase in the numbers of American hunters during the 1990s. The marked change in the proportions of black duck harvested in the two jurisdictions is therefore, at least in part, simply a reflection of changes in the relative abundances of hunters. The decrease was also concurrent with the deployment of the zip code bands. Previous studies had suggested that the implementation of the toll-free lines and the web application was more readily adopted by American hunters than by Canadian hunters (Zimmerman et al. 2009; Boomer et al. 2013; Garrettson et al. 2013), so it is not impossible that zip code bands were also more readily adopted by American hunters. Adding the ZIP code should have helped American hunters in determining where to report their bands, but this information would not have necessarily helped a Canadian hunter who would be more familiar with the postal codes used in Canada. Managers speculate that language impediments experienced by francophone hunters are partly responsible for the discrepancy in reporting rate between the two countries (Zimmerman et al. 2009; Boomer et al. 2013; Garrettson et al. 2013). This has been disputed by Souchay et al. (2014), who did not find any differences in reporting rate of Greater Snow Geese (*Chen caerulescens atlantica*) bands between Quebec and the Eastern USA. However, we detected a slight increase in the proportion of bands reported in Canada subsequent to the deployment of the web bands. This effect could lend some support to the hypothesis that francophones black duck hunters did not use the toll-free line as readily as other groups.

### Spatial variation in harvest probabilities

Within Canada, the highest 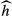 were found near populated areas. This may be a reflection of the distribution of the harvest effort in Canada, an explanation supported by the strong spatial gradient that we observed in the proportion of black duck harvested in Canada. High values of 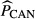 in southern blocks imply that Canadian hunters harvest ducks from the local populations rather than northern migrants. Ducks have been shown to be highly vulnerable during the first few days of the hunting season and most hunters report their kills early in the season (Martin and Carney 1977; Parker 1991; Longcore et al. 2000a). In southern Quebec, the majority of the harvest occurs during the first 3 weeks of the season, a period during which ducks breeding in the northern boreal habitat are unlikely to have initiated their fall migration (Cousineau et al. 2014). These results imply that Canadian and American hunters hunt different populations. This could represent a challenge to the realization of current management objectives, because national managers would then have conflicting interests in terms of the spatial focus of habitat and population management efforts.

The pattern of spatial random effects indicated that 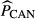 was correlated with migratory pathways. Black ducks associated with the Western management unit were less likely to be harvested in Canada than those associated with the Central management unit (Fig.1A). Black ducks originating from northern Ontario could be avoiding the high hunting pressure in the south or they could be simply using a migratory pathway inaccessible to hunters (Ashley et al. 2010; Szymanski et al. 2013). In contrast, birds originating from the Central management unit migrate along the Saint Lawrence estuary and river system where the population of hunters is concentrated. Black ducks migrating on this route are therefore exposed to higher risk of harvest (Reed and Boyd 1974) and are consequently more prone to be harvested in Canada.

### Temporal variation in harvest probability

Harvest probabilities declined over the study period, but the magnitude of decline did not meet the NAWMPS target of 25%. This seems to be because the variation in *h* for juveniles, who represent the bulk of the harvest, was more closely tied to hunting effort than to regulation. Previous efforts to identify the effect of the more restrictive regulations (Krementz et al. 1987; Francis et al. 1998) also tended to favor a decrease in hunting pressure, which is consistent with our findings. We could not accurately measure how *h* evolved through time, as we had no reliable way to estimate annual *λ*. It is thus possible that *h* were low during the 1980s, enabling the population to stabilize, while the subsequent lack of growth is due to the increase of American hunters in the 1990s. Such explanations cannot be tested with the present dataset. Our comparison of 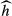 between 1982 and 2010 hinge on the accuracy of the λ studies. Conroy and Blandin (1984) had limited data and managers have often speculated that the lack of power of the analysis prevented the authors from finding a difference between Canada and the USA. However, λ were lower in the 1980s than currently, and the information on the avise bands was rather cryptic, so the difference in λ was probably not as marked then as it is today. It's much more likely that the λ diverged between Canada and the U.S. over time with the addition of information on the bands, the campaign of information about the banding program and the occasional band reward programs.

Despite declining harvest probabilities in the north, southern populations remain under high pressure (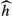 > 0.20) particularly along the Saint Lawrence River system and in the Atlantic Provinces. Reed and Boyd (1974) estimated *h* for juvenile black ducks to be from 0.20 to 0.40 in the Saint Lawrence estuary, consistent with our findings. While it is generally agreed that hunting mortality is compensatory rather than additive below a certain threshold, it is still possible for kill probabilities to exceed such a threshold locally (Anderson and Burnham 1976). As the compensation threshold for ducks is probably low, the assumption of additive mortality risks is conservative, in the absence of good reasons to believe otherwise (Lebreton 2005). As the populations in the south are much smaller than those in the north, the current rate of harvesting might be unsustainable (Cousineau et al. 2014). We recommend that the southern populations we identified be monitored more closely.

The most severe black duck population declines have been observed on the Mississippi flyway, whose migrants typically come from Ontario and western Quebec (Link et al. 2006). Our derived hunting probabilities for populations breeding in the northwestern breeding area of Canada were low; however, the Risk ratio indicated that the decline in *h* reached their highest values in this region (Risk ratio < 0.80). This suggests that the population declines observed in those regions are probably not the result of overharvesting.

## Conclusion

The marked spatial variation in *h* we identified leads us to conclude that more needs to be done to elucidate the fine spatial structure of harvest in black duck population. Harvesting probabilities remain high for southern populations. Longcore et al. (2000a) had advocated the creation of nonhunting zones along the Saint Lawrence estuaries. These could diminish the pressure on local populations. Revisiting this idea in the southern breeding range of the black duck could be an effective way to manage populations without imposing complex regulations. We also have poor knowledge of the populations in the north, which hampers our ability to properly estimate current harvest probabilities. Given their potential importance to harvest dynamics between the two countries, and the objectives to maintain a balanced harvest between Canada and the USA (Conroy and Peterson 2012), we recommend that more attention be given to the northern populations. Our results highlight the importance of accurate knowledge of reporting probabilities in estimating harvest probabilities. Given that λ will probably continue to evolve through time, it is important they continue to be monitored closely. Finally, given the spatial differences, we identified in *h* the next step would be made to develop spatially explicit recruitment and survival models to quantify how the local populations are affected by the current levels of harvesting.

## Supporting Information

**Appendix S1.** Spatiotemporal Stan model for direct recoveries
