# Supplementary material for: Spatial and temporal variation in harvest probabilities for American black duck
Source: Ecol Evol. 2015 Apr 16;5(10):1992–2004. doi: 10.1002/ece3.1484 (PMC4449754; doi:10.1002/ece3.1484)
Supplement: Supplementary file 1 [file ece30005-1992-sd1.docx]

# Supporting Information

## Appendix S1: Spatio-temporal Stan model for direct recoveries

data {

int<lower=1> Ncells; //Number of banding blocks

int<lower=1> Year; //Number of years

int Y1_ad[Ncells,Year]; //Matrix holding the numbers of banded adults

int Y2_ad[Ncells,Year]; //Matrix holding the numbers of banded adults

int Y3_ad[Ncells,Year]; //Matrix holding the numbers of banded adults

int Y4_ad[Ncells,Year]; //Matrix holding the numbers of banded adults

int N1_ad[Ncells,Year] ; //Matrix holding the numbers of adults direct recoveries

int N2_ad[Ncells,Year]; //Matrix holding the numbers of adults direct recoveries

int N3_ad[Ncells,Year]; //Matrix holding the numbers of adults direct recoveries

int N4_ad[Ncells,Year]; //Matrix holding the numbers of adults direct recoveries

int Y1_im[Ncells,Year]; //Matrix holding the numbers of banded immature

int Y2_im[Ncells,Year]; //Matrix holding the numbers of banded immature

int Y3_im[Ncells,Year]; //Matrix holding the numbers of banded immature

int Y4_im[Ncells,Year]; //Matrix holding the numbers of banded immature

int N1_im[Ncells,Year]; //Matrix holding the numbers of immature direct recoveries

int N2_im[Ncells,Year]; //Matrix holding the numbers of immature direct recoveries

int N3_im[Ncells,Year]; //Matrix holding the numbers of immature direct recoveries

int N4_im[Ncells,Year]; //Matrix holding the numbers of immature direct recoveries

int<lower=1> D; //numbers of covariables

real min_theta; //lower bound prior theta

real max_theta; //upper bound prior theta

vector[Ncells] mvn0; //vector 0 for mnvorm

vector[2] coords[Ncells]; //2 colum matrix holding the banding blocks centroids

row_vector[D] X[Year]; //Covariable matrix

}

parameters {

vector[2] alpha1; // mean avise

vector[2] alpha2; // mean zip

vector[2] alpha3; // mean toll

vector[2] alpha4; // mean web

corr_matrix[2] Rho_alpha;

vector<lower=0>[2] sigma_alpha; vector[D] beta_ad[1];

vector[D] beta_im[1];

//temporal error

real<lower=0, upper=20> sigma_epsilon;

vector[Ncells] epsilon_ad[Year];

vector[Ncells] epsilon_im[Year];

//Spatial error

real<lower=0, upper=20> sigma_spatial;

//Covariance

real<lower=min_theta, upper=max_theta> theta;

vector[Ncells] Z_prime;

}

transformed parameters{

//Covariance Matrix

corr_matrix[Ncells] K;

cov_matrix[Ncells] Omega;

real variance_spatial;

real Z_mu;

vector[Ncells] Z;

//likelihood

vector[Ncells] logit_prob_ad1[Year];

vector[Ncells] logit_prob_ad2[Year];

vector[Ncells] logit_prob_ad3[Year];

vector[Ncells] logit_prob_ad4[Year];

vector[Ncells] logit_prob_im1[Year];

vector[Ncells] logit_prob_im2[Year];

vector[Ncells] logit_prob_im3[Year];

vector[Ncells] logit_prob_im4[Year];

//Intercept

Omega_alpha <- quad_form_diag(Rho_alpha,sigma_alpha);

//Spatial covariance

variance_spatial <- pow(sigma_spatial,2);

for (i in 1:Ncells) {

for (j in 1:Ncells) {

K[i,j] <- exp(-distance(coords[i],coords[j])*theta);

}}

Omega <- variance_spatial*K;

//Re-center Z to insure identifiability

Z_mu <- mean(Z_prime);

Z <- Z_prime - Z_mu;

//Estimate prob on logit scale

for(t in 1:Year){

logit_prob_ad1[t] <- alpha1[1] + X[t]*beta_ad[1] + Z + epsilon_ad[t]*sigma_epsilon;

logit_prob_ad2[t] <- alpha2[1] + X[t]*beta_ad[1] + Z + epsilon_ad[t]*sigma_epsilon;

logit_prob_ad3[t] <- alpha3[1] + X[t]*beta_ad[1] + Z + epsilon_ad[t]*sigma_epsilon;

logit_prob_ad4[t] <- alpha4[1] + X[t]*beta_ad[1] + Z + epsilon_ad[t]*sigma_epsilon;

logit_prob_im1[t] <- alpha1[2] + X[t]*beta_im[1] + Z + epsilon_im[t]*sigma_epsilon;

logit_prob_im2[t] <- alpha2[2] + X[t]*beta_im[1] + Z + epsilon_im[t]*sigma_epsilon;

logit_prob_im3[t] <- alpha3[2] + X[t]*beta_im[1] + Z + epsilon_im[t]*sigma_epsilon;

logit_prob_im4[t] <- alpha4[2] + X[t]*beta_im[1] + Z + epsilon_im[t]*sigma_epsilon;

} }

model{

// mean

alpha1 ~ multi_normal(mvn_alpha, Omega_alpha);

alpha2 ~ multi_normal(mvn_alpha, Omega_alpha);

alpha3 ~ multi_normal(mvn_alpha, Omega_alpha);

alpha4 ~ multi_normal(mvn_alpha, Omega_alpha);

sigma_alpha ~ cauchy(0,2.5);

Rho_alpha ~ lkj_corr(1);

//Explanatory variables

sigma_beta ~ cauchy(0,1);

for(i in 1:D){

beta_ad[1,i] ~ normal(0,sigma_beta[i]);

beta_im[1,i] ~ normal(0,sigma_beta[i]);

}

//Spatial error

sigma_spatial ~ cauchy(0,1);

//Spatial Covariance

theta ~ uniform(min_theta,max_theta);

Z_prime ~ multi_normal(mvn0, Omega);

//temporal and small scale error

sigma_epsilon ~ cauchy(0,1);

for(t in 1:Year){

epsilon_ad[t] ~ normal(0,1);

epsilon_im[t] ~ normal(0,1);

}

//Likelihood

for(i in 1:Ncells){

for(t in 1:Year){

Y1_ad[i,t] ~ binomial_logit(N1_ad[i,t], logit_prob_ad1[t,i]);

Y2_ad[i,t] ~ binomial_logit(N2_ad[i,t], logit_prob_ad2[t,i]);

Y3_ad[i,t] ~ binomial_logit(N3_ad[i,t], logit_prob_ad3[t,i]);

Y4_ad[i,t] ~ binomial_logit(N4_ad[i,t], logit_prob_ad4[t,i]);

Y1_im[i,t] ~ binomial_logit(N1_im[i,t], logit_prob_im1[t,i]);

Y2_im[i,t] ~ binomial_logit(N2_im[i,t], logit_prob_im2[t,i]);

Y3_im[i,t] ~ binomial_logit(N3_im[i,t], logit_prob_im3[t,i]);

Y4_im[i,t] ~ binomial_logit(N4_im[i,t], logit_prob_im4[t,i]);

}}

}
